# Circ-APBB1IP as a Prognostic Biomarker Promotes Clear Cell Renal Cell Carcinoma Progression Through The ERK1/2 Signaling Pathway

**DOI:** 10.7150/ijms.44550

**Published:** 2020-05-18

**Authors:** Jierong Mo, Yuwan Zhao, Zhixian Ao, Lixin Chen, Shanhong Lin, Wenfeng Zeng, Haokai Wu, Jianjun Liu

**Affiliations:** Laboratory of Urology, Affiliated Hospital of Guangdong Medical University, Zhanjiang, Guangdong 524001, China

**Keywords:** circ-APBB1IP, clear cell renal cell carcinoma, progression, ERK1/2 signaling pathway

## Abstract

Circular RNA (circRNA), a member of non-coding RNA, plays an essential regulatory role in many human physiological and pathological processes; however, its role in clear cell renal cell carcinoma (ccRCC) still unclear. This study aims to investigate the effect and mechanisms of circRNA on ccRCC progression. A human circRNA microarray was used to discover differential expression circRNA, and a quantitative real-time polymerase chain reaction (qRT-PCR) was performed to verify the expression of circRNA. The function and mechanism of circRNA were explored by cell transfection, cell counting kit-8, fluorescein isothiocyanate (FITC) Annexin V apoptosis detection, wound healing, transwell, and western blot. The result indicated that circ-APBB1IP was significantly up-regulated in ccRCC. In vitro, knockdown of circ-APBB1IP by siRNA suppressed the proliferation, migration, and invasion and increased the apoptosis of ccRCC cells. Further study found that knockdown of circ-APBB1IP up-regulated protein expression of cleaved caspase-3, cleaved caspase-7, cleaved caspase-8, cleaved caspase-9, Bax, Bad, Bak, E-cadherin and down-regulated expression of Bcl-2, N-cadherin, MMP-2, MMP-9, p-ERK1/2. Our result indicates that circ-APBB1IP has a vital function in ccRCC tumorigenesis. These findings suggest that circ-APBB1IP represents a novel potential biomarker and therapeutic target of ccRCC.

## Introduction

Clear cell renal cell carcinoma (ccRCC) is the most common renal cell carcinoma, and approximately 30% of ccRCC patients have metastatic disease at the time of diagnosis 1. However, the pathogenesis of ccRCC is not extremely clear. At present, Partial nephrectomy is usually the standard treatment for non-metastatic clear cell carcinoma with good results 2. Nevertheless, patients with metastatic renal cell carcinoma have a poor prognosis and a 5-year survival rate of only 10% 3. Therefore, it is urgent to seek valid therapeutic targets.

Circular RNA (circRNA) is one of the novel noncoding RNA with various molecular functions. Different from micro RNA (miRNA) and long non-coding RNA (lncRNA), circRNA has a unique closed-loop structure 4, 5. Due to without 5' cap or 3' poly (A) tail, circRNA is more stable and resistant than linear RNA 6. Recently, circRNA become a hot molecule in cancer progression research; the progression of tumor growth, apoptosis, metastasis, and drug resistance have been reported to get closely related to circRNA 7. For example, circular RNA ABCB10 was related to apoptosis in ccRCC cells 8. Circ-LDLRAD3 promotes the proliferation, migration, and invasion of pancreatic cancer cells 9. FBXW7 circular RNA regulates proliferation, migration, and invasion of colorectal carcinoma through NEK2, mTOR, and PTEN signaling pathways 10. Circ-0000284 arouses the malignant phenotype of cholangiocarcinoma cells and regulates the biological functions of peripheral cells through cellular communication 11. CircRNA can be used as a diagnostic target and biomarker for diseases because of its diagnostic and therapeutic potential in many diseases 12.

In this present study, human circRNA microarray analysis was performed to screen circRNA expression profiles in ccRCC cancer tissue. According to the cutoff values (fold change > 1.5 and P < 0.05) to filter differential expression circRNA, there were 356 differential expressions circRNA were detected, of which 127 circRNA were up-regulation, and 229 circRNA were down-regulation, circRNA with high fold change were selected to verification. The result showed that circ-APBB1IP was significantly overexpression in ccRCC tissues. The aim of this study was to verify whether circ-APBB1IP plays a regulatory role in the progression of ccRCC.

## Materials and Methods

### Patient tissue samples and cell culture

A total of 14 pairs of tumor tissues and tumor-distant tissues of ccRCC were collected from the Affiliated Hospital of Guangdong Medical University, and three pairs were used to perform microarray analysis. In comparison, 11 pairs were used to perform qRT-PCR. All tissue samples have been identified by pathologists in this hospital. This study was approved by the Ethics Committee of Affiliated Hospital of Guangdong Medical University. All samples were immediately frozen in liquid nitrogen after resection and stored at -80°C until use. Human ccRCC cells (786-O and Caki-1) were obtained from the cell bank of the Chinese Academy of Science (Shanghai, China), and the normal renal cell line (HK-2) was obtained from Guangzhou Jennio Biological Technology Co., Ltd. (Guangzhou, China). 786-O cells were cultured in Roswell Park Memorial Institute-1640 medium (RPMI 1640 medium) (GIBCO, Thermo Fisher Scientific, Inc., Waltham, MA, USA), while Caki-1 cells were cultured in McCoy's 5A medium (Sigma, Merck Life Science, (Shanghai) Co. Ltd), HK-2 cells were grown in Dulbecco's modified Eagle's medium (DMEM) (GIBCO, Thermo Fisher Scientific, Inc., Waltham, MA, USA). All culture mediums were supplemented with 10% (v/v) fetal bovine serum (FBS; GIBCO, Thermo Fisher Scientific, Inc., Waltham, MA, USA) at 37 °C in a humidified atmosphere that contained 5% CO_2_.

### Human circRNA microarray analysis

Arraystar Human circRNA Array V2 was used to analyze three pairs of ccRCC tissues. Total RNA from each sample was quantified using the NanoDrop ND-1000 and digested using Rnase R (Epicentre, Inc., WI, USA) to remove linear RNAs and enrich circular RNAs. Then, the enriched circular RNAs were amplified and transcribed into fluorescent cRNA (Arraystar Super RNA Labeling Kit; Arraystar). The labeled cRNAs were hybridized onto the Arraystar Human circRNA Array V2 (8×15K, Arraystar). The arrays were scanned by the Agilent Scanner G2505C (Agilent Technologies, Jamul CA, USA).

### Quantitative real-time polymerase chain reaction (qRT-PCR)

Total RNA was extracted from the tissue samples and cultured cells using TRIzol reagent (Invitrogen, Carlsbad CA, USA). According to the manufacturer's instructions, using the PrimeScript™ RT reagent Kit with gDNA Eraser (Takara, Japan) to synthesize cDNA through reverse transcribed. Then, we performed qRT-PCR using TB Green® Premix Ex Taq™ (Tli RNaseH Plus) (Takara, Japan) according to the manufacturer's instructions including an initial denaturation step (95°C for 30 s) and 40 cycles of denaturation (95°C for 5 s) and annealing (60°C for 30 s). The qRT-PCR process was conducted on the Light Cycler 480 II Real-Time PCR system. The result was calculated using the 2^-ΔΔCt^ method, and GAPDH was used as an internal reference 13, 14. The sequence of the primer is as follows: circ-APBB1IP: forward: 5′-TCAGACTGGACAAGAGACACAGA-3′, reverse, 5′-TGTCCTCTGCTCAGCCTCAC-3′; GAPDH: forward: 5′-GGTGAAGGTCGGAGTCAACGG-3′, reverse, 5′-CCTGGAAGATGGTGATGGGATT-3′. Primers for circ-APBB1IP and GAPDH were synthesized by Sangon Biotech (Shanghai, China).

### Cell transfection

According to the manufacturer's instructions, cells of ccRCC were transfected with lipofectamine 3000 (Invitrogen, USA). The sequence of siRNA (sense: AAACCCCCAGAGUCCUUAATT, antisense: UUAAGGACUCUGGGGGUUUTT) and negative control (NC) (sense: UUCUCCGAACGUGUCACGUTT, antisense: ACGUGACACGUUCGGAGAATT) were designed and synthesized by GenePharma Co., Ltd. (Shanghai, China). Three μl lipofectamine 3000 and 3 μl siRNA (20 μM) were diluted in 125 μl Opti-MEM medium without supplement respectively 5 minutes, as well as NC group was performed with the same formulation, then, cells were incubated with siRNA-lipofectamine complexes for 6 hours and were cultured in 6-well plates (Nest Biotechnology, Wuxi, China) for 24 hours. In order to verify whether the transfection was successful, the expression of circ-APBB1IP was detected by qRT-PCR after transfections. The independent experiment was repeated three times.

### Cell proliferation assay

Cell Counting Kit-8 (CCK8) (Apexbio, HOU USA) assay was used to investigate the proliferation of ccRCC cells. The cells of group NC and group SI- circ-APBB1IP were cultured in 96-well plate (3000 cells/well) (Nest Biotechnology, Wuxi, China), Then, the cells of every well added ten μl CCK8 and detected optical density (OD) value after 2 hours. The OD value was detected every 24 hours. The absorbance of the solution at 450 nm was detected using a Multiskan Ascent microplate photometer (EnSpire 2300 Multilabel Reader, PE, USA).

### Flow cytometry

The apoptosis of cells was detected using Fluorescein Isothiocyanate (FITC) Annexin V Apoptosis Detection Kit (BD Biosciences, Franklin Lakes, NJ, USA). Cells of 786-O and Caki-1 were digested after transfection, then washed with PBS, and resuspended in the binding buffer. FITC-labeled annexin V and propidium iodide were used to stain cells for 15min. Finally, the analysis of stained cells was performed using the flow cytometry BD FACSDiva 6.1 software.

### Wound healing (migration) assay and transwell (invasion) assay

The migratory ability of ccRCC cells was assessed using a Wound healing (migration) assay. 786-O and Caki-1 cells were seeded into 6-well plates after cell transfection when the cells fill the 6-well plates, a pipette tip was used to make scratches in the monolayer of ccRCC cells, the floating cells were washed off with PBS, and then cells in 6-well plates were cultured with medium without FBS. The migration of 786-O cell was observed after 12 hours, while Caki-1 cell was recorded after 48 hours. The images were taken with EVOS XL Core Imaging System (Life Technologies, USA). The invasive ability of ccRCC cells was detected using the transwell assay. After transfection, 786-O cell (5×10^4^ cells/well) and Caki-1 cell (1×10^5^ cells/well) were resuspended in 200 μl serum-free medium and planted them in the upper chamber of the insert (membranepore size, eight μm; Corning) with Matrigel (BD Biosciences, Billerica, MA, USA). There was 750 μl of 20% FBS-medium in the bottom chambers. After 24 hours of culture, the cells adhering to the base membrane of the inserts were detected with EVOS XL Core Imaging System.

### Western blot analysis

The total proteins were extracted with RIPA buffer (Beyotime, Shanghia, China) supplemented with 1 mM PMSF (phenylmethanesulfonyl fluoride) (Beyotime, Shanghai China) either 48 hours after transfection. All proteins were separated by SDS-PAGE and transferred onto a PVDF membrane (EMD Millipore, Billerica, MA, USA). The membrane was incubated with primary antibody at 4℃ overnight after blocked with 5% non-fat milk. Primary Antibody Dilution Buffer (Beyotime) were used to dilute primary antibody cleaved caspase-3 (#9665S; dilution 1: 1,000), cleaved caspase-7 (#9494S; dilution 1: 1,000), cleaved caspase-8 (#4790S; dilution 1: 1,000), cleaved caspase-9 (#9502S; dilution 1: 1,000), Bax (#2772S; dilution 1: 1,000), Bad (#9239; dilution 1: 1,000), Bak (#12105S; dilution 1: 1,000), Bcl-2 (#2876S; dilution 1: 1,000), E-cadherin (#3195S; dilution 1: 1,000), N-cadherin (#4061S; dilution 1: 1,000), MMP-2 (#87809S; dilution 1: 1,000), MMP-9 (#2270S; dilution 1: 1,000), ERK1/2 (#4695S; dilution 1: 1,000), p-ERK1/2 (#4370S; dilution 1: 1,000) (all Cell Signaling Technology, Inc., Danvers, MA, USA). Then wash three times with Tris-buffered saline and Tween 20 (TBST) for 10 minutes, and were incubated with IgG-HRP secondary antibody (EarthOx, USA) for 1hour at room temperature. The protein bands were detected using an enhanced chemiluminescence kit (EMD Millipore, Billerica, MA, USA) with Tanon 5200 chemiluminescent imaging system (Shanghai, China).

### Statistical analysis

All data were analyzed with GraphPad Prism 7 (GraphPad Software, La Jolla, CA, USA), and the results were expressed as the mean ± SD. The differences between groups were determined by unpaired Student's t-test analysis. A value of *P* < 0.05 was considered statistically significant.

## Results

### The expression of circ-APBB1IP in clinical CRC patients and ccRCC cells

**Figure 1:**
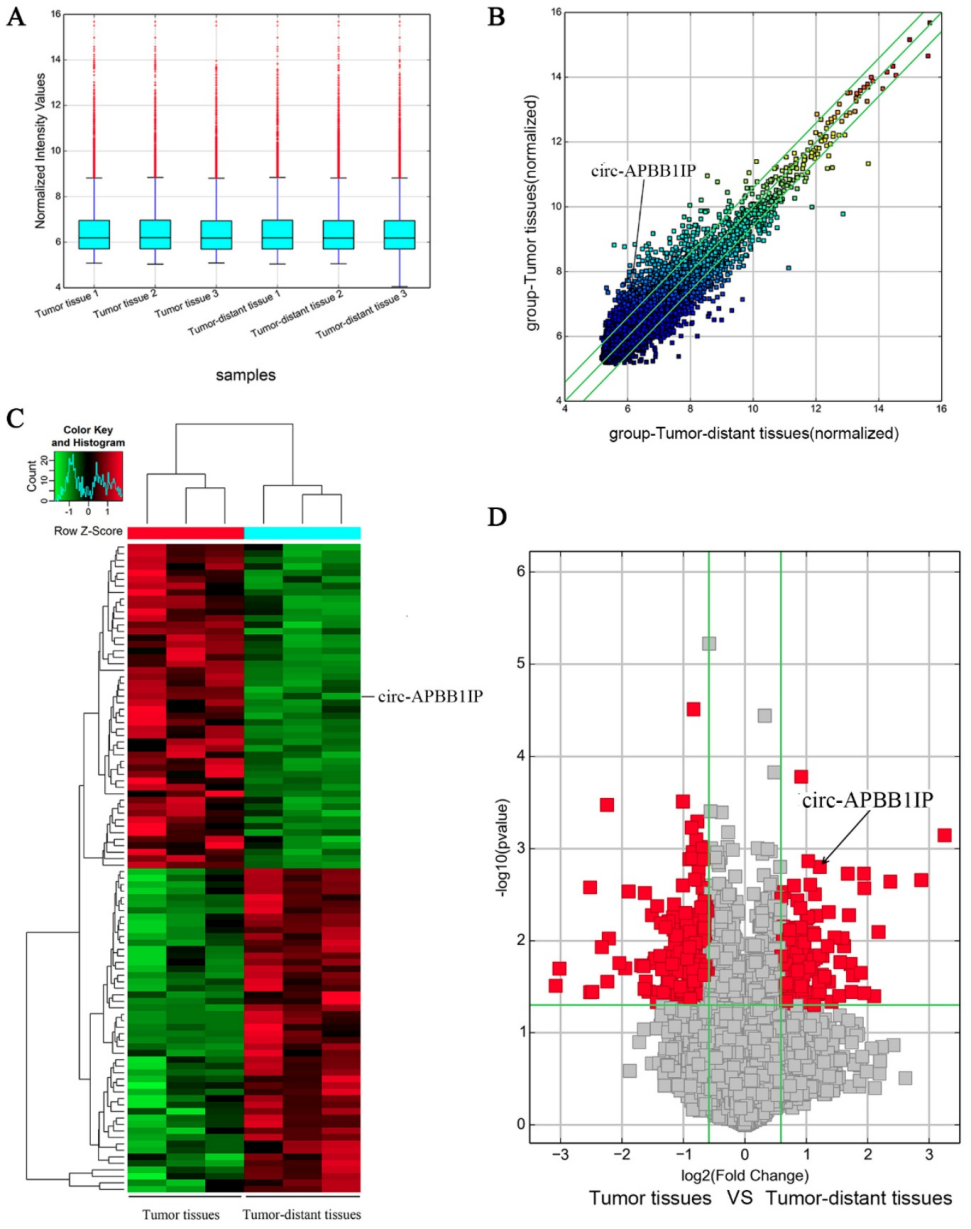
CircRNA expression profile in clear cell renal cell carcinoma tissue. **(A)** Box plot showed the normalized intensities from the tumor and tumor-distant tissues samples.** (B, D)** Scatter plots and volcano plots showed the visualizing circRNA different expression in clear cell renal cell carcinoma tumor tissues and tumor-distant tissues. **(C)** The heat map showed the selected 50 upregulated (red) and 50 downregulated (green) circRNA.

**Figure 2:**
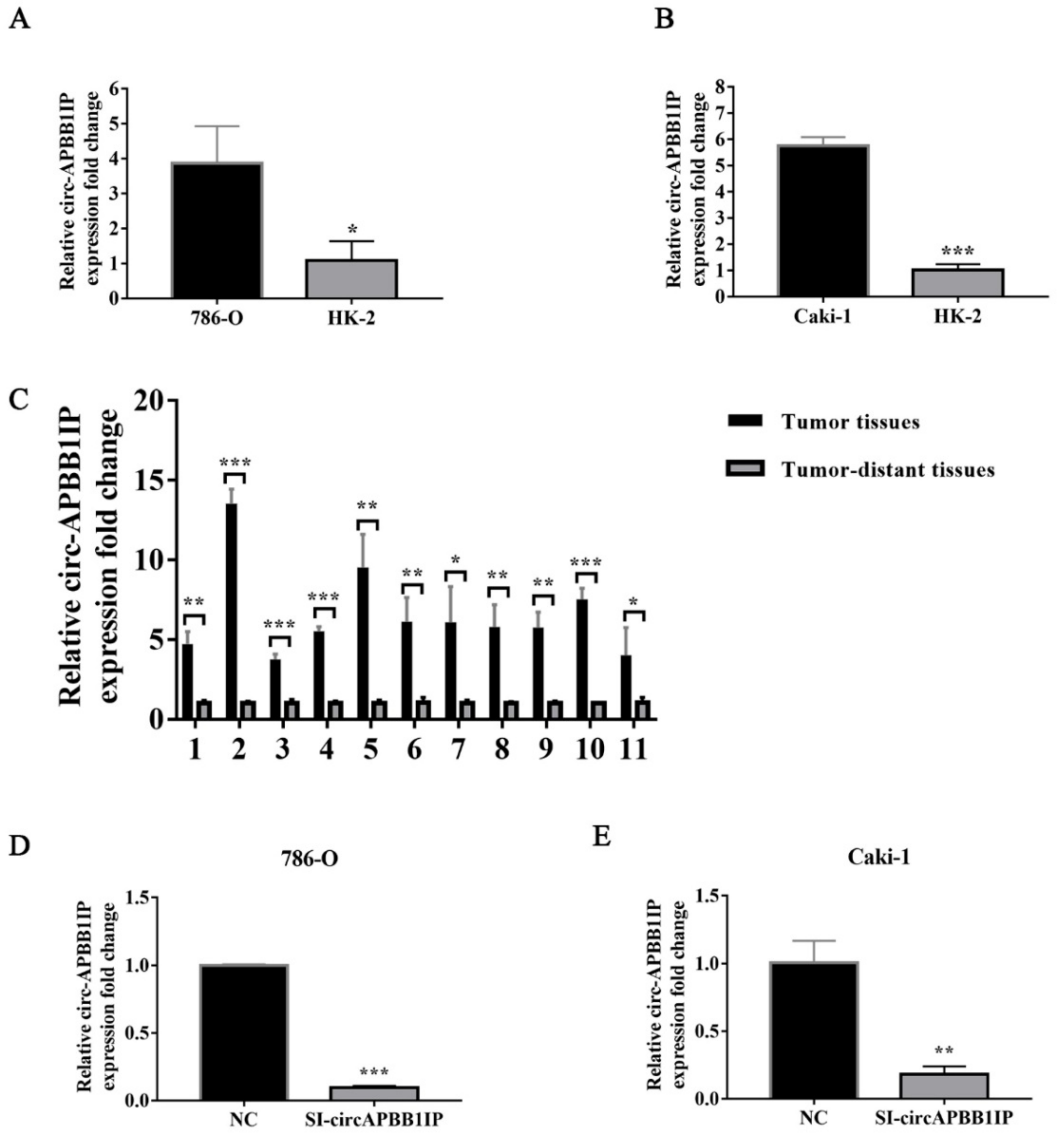
Circ-APBB1IP was upregulated in 11 ccRCC tissue samples and cell lines. **(A, B)** qRT-PCR was used to determine the circ-APBB1IP expression level in 786-O and Caki-1 cell lines. **(C)** The circ-APBB1IP expression level in 11 pairs ccRCC patient tissues was quantified using qRT-PCR. Circ-APBB1IP was knockdown in ccRCC cell lines using a specific siRNA targeting circ-APBB1IP. The expression levels of circ-APBB1IP were explored using qRT-PCR after Transfections in 786-O **(D)** and Caki-1 **(E)** cells. The data are presented as the mean ± SD for three independent experiments. T-test, *P < 0.05, **P < 0.01, and ***P < 0.001.

We used three pairs of ccRCC tumor tissues and tumor-distant tissues to screen the expression of circRNA using human circRNA microarray. The normalized intensities of tumor tissues and tumor-distant tissues samples were shown in Box plots (Fig. 1A). Scatter plots and volcano plots showed the differential expression of circRNA in ccRCC tumor tissues and tumor-distant tissues (Fig. 1B and 1D). The heat map consisted of 100 genes that were significantly up-regulated and down-regulated (Fig. 1C). This different expression circRNA might play a critical role in the development and progression of tumors. After screening, we focused on the circ-APBB1IP (circbase ID: hsa_circ_0093390), which located in chr10:26789747-26802589. The expression of circ-APBB1IP was further verified in ccRCC cell lines and patient tissues. The expression of circ-APBB1IP in ccRCC cell lines 786-O and Caki-1 was significantly higher than that in normal renal cell line HK-2 (Fig. 2A and 2B), and a similar pattern was seen in 11 pairs ccRCC tumor tissues and tumor-distant tissues (Fig. 2C). These data showed that circ-APBB1IP might be related to the development and progression of ccRCC.

### Knockdown of circ-APBB1IP of ccRCC cells

The expression of circ-APBB1IP was up-regulated in ccRCC cells, in order to further investigate the role of circ-APBB1IP in ccRCC, we used a specific siRNA targeting circ-APBB1IP to knockdown the expression of circ-APBB1IP in 786-O and Caki-1 cells. The expression of circ-APBB1IP decreased obviously (Fig. 2D and 2E).

### The knockdown of circ-APBB1IP inhibited the proliferation and promoted the apoptosis of ccRCC cells

**Figure 3:**
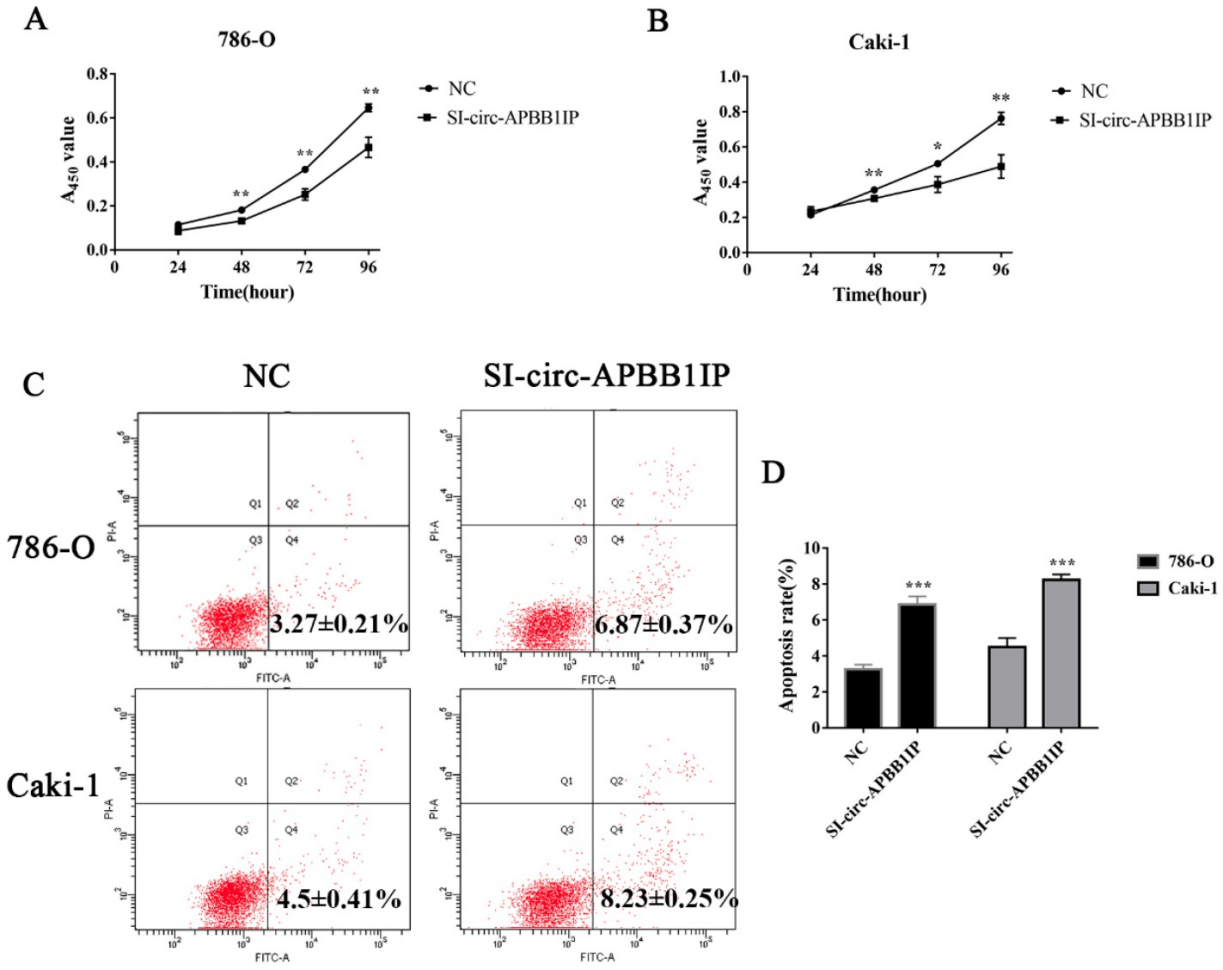
The knockdown of circ-APBB1IP inhibited proliferation and promoted apoptosis in ccRCC cells. **(A, B)** The CCK-8 assay was performed to determine cell proliferation at different time points after infection. **(C, D)** Fluorescein isothiocyanate (FITC) Annexin V Apoptosis Detection kit was used to determine the apoptotic rate in 786-O and Caki-1 cells. The data are presented as the mean ± SD for three independent experiments. T-test, *P < 0.05, **P < 0.01, and ***P < 0.001.

To explore the role of circ-APBB1IP in regulating ccRCC cells proliferation and apoptosis, CCK-8 assay was used to measure the proliferation of ccRCC cells. The proliferation of 786-O and Caki-1 was significantly inhibited after silencing circ-APBB1IP (Fig. 3A and 3B). Besides, the apoptosis rate of 786-O and Caki-1 cells was detected by flow cytometry. The results showed that the apoptosis rate of the cells increased significantly after knockdown circ-APBB1IP (Fig. 3C and 3D).

### The knockdown of circ-APBB1IP inhibited the migration and invasion of ccRCC cells

**Figure 4:**
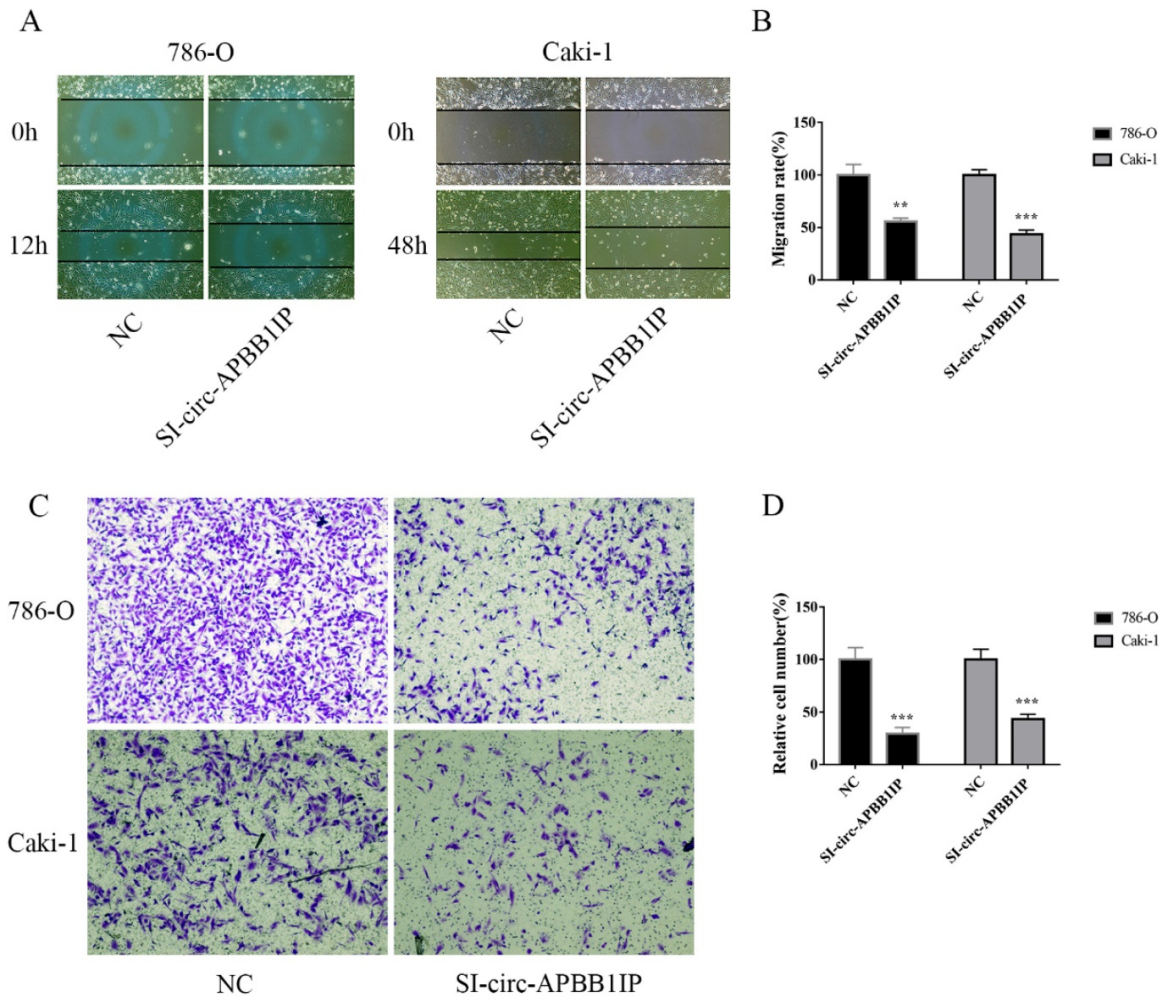
The knockdown of circ-APBB1IP inhibited migration and invasion in ccRCC cells. **(A, B)** The wound healing (migration) assay was used to measure the migration rate of ccRCC cells, the migration rate of 786-O was detected at 12 h after the scratch, while Caki-1 at 48 h after the scratch.** (C, D)** Transwell assay was performed to determine the invasion rate of ccRCC cells, 786-O cell (5×10^4^ cells/well), and Caki-1 cell (1×10^5^ cells/well) were seeded into the Matrigel-coated upper chamber, after 24 h, those that passed across the coated chamber were detected. The data are presented as the mean ± SD for three independent experiments. T-test, **P < 0.01, and ***P < 0.001

To investigate the possible mechanism of circ-APBB1IP in ccRCC, circ-APBB1IP was knockdown by a specific siRNA targeting circ-APBB1IP. The migration ability of 786-O and Caki-1 cells was measured by wound healing (migration) assay. Comparing with control, the migration of 786-O and Caki-1 cells was suppressed significantly (Fig. 4A and 4B). Also, we used the transwell assay to detect the invasion ability of 786-O and Caki-1 cells. The results showed that the invasion of 786-O and Caki-1 cells was inhibited prominently after knockdown the expression of circ-APBB1IP (Fig. 4C and 4D).

### Circ-APBB1IP regulated tumor growth through the ERK1/2 signaling pathway

**Figure 5:**
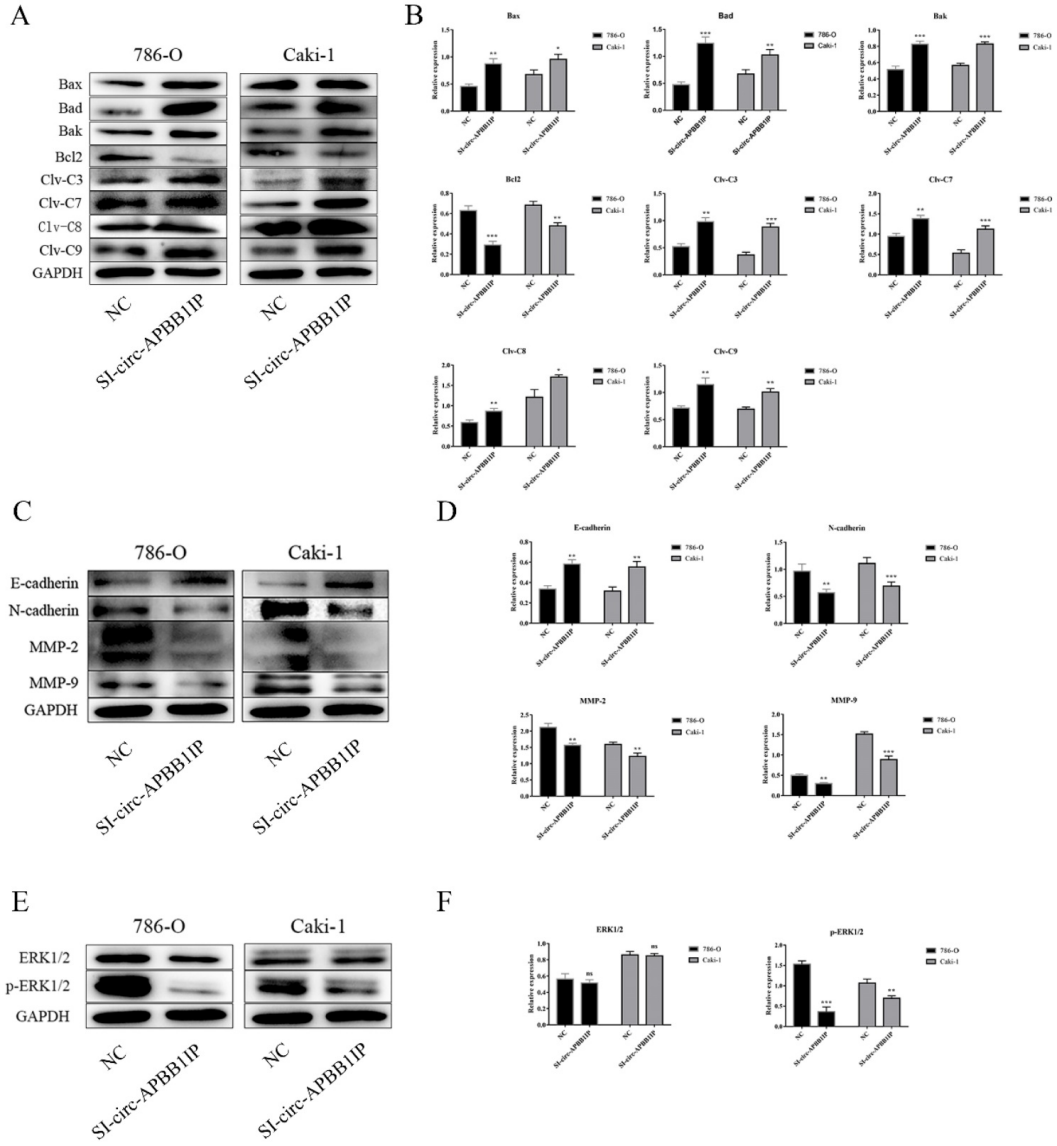
The knockdown of circ-APBB1IP affected the levels of key proteins involved in cell apoptosis, migration, invasion, and the ERK1/2 signaling pathway. **(A, B)** Expression levels of apoptosis-related protein (Bax, Bad, Bak, Bcl-2, cleaved caspase 3, cleaved caspase 7, cleaved caspase 8, cleaved caspase 9), **(C, D)** migration-related protein (MMP-2, MMP-9) and invasion-related protein (E-cadherin, N-cadherin) were measured by Western blot assay. **(E, F)** The ERK1/2 signaling pathway markers were determined by western blot. The data are presented as the mean ± SD for three independent experiments. T-test, ns *p* > 0.05, *P < 0.05, **P < 0.01, and ***P < 0.001.

To further determine the potential molecular mechanism of circ-APBB1IP in regulating ccRCC cell growth. Western blot assay was performed to measure the expression of related proteins. Caspases, a family of cysteine proteases, has the function of regulating apoptosis, they produce cleaved caspase after stimulation 15. Pro-apoptotic proteins Bak, Bad, Bid, Bax and anti-apoptotic proteins Bcl-2, belong to Bcl-2 family proteins, play a critical role in apoptosis 16. The result showed that knockdown the expression of circ-APBB1IP, the expression of pro-apoptotic protein such as cleaved caspase-3, cleaved caspase-7, cleaved caspase-8, cleaved caspase-9, Bax, Bad, Bak were up-regulated. In contrast, the anti-apoptotic protein (Bcl-2) was down-regulated in the cells of silence circ-APBB1IP compared with the negative control (Fig. 5A and 5B). Besides, Cadherin regulates the progression of invasion in cancer, E-cadherin inhibits invasion, while N-cadherin promotes invasion 17. Matrix metalloproteinases (MMPs) get closely related to tumor metastasis, which permits cells to traverse the ECM to reach distant position 18. To explore the role of circ-APBB1IP in the migration and invasion of ccRCC cells, we measured E-cadherin, N-cadherin, MMP-2, MMP-9. Knockdown the expression of circ-APBB1IP, the result of western blot assay showed significant up-regulation in E-cadherin expression, but the reduction of N-cadherin, MMP-2 and MMP-9 (Fig. 5C and 5D). Besides, ERK1/2 signaling pathway regulates many cellular functions, such as apoptosis, proliferation, migration and invasion 19-21. To explore the character of the ERK1/2 signaling pathway, we detected the expression of ERK1/2 and p-ERK1/2 using western blot assay. The data showed that the expression of p-ERK1/2 was reduced significantly after knocking down circ-APBB1IP (Fig. 5E and 5F).

## Discussion

The incidence and mortality rates of ccRCC are increasing though the methods of diagnosis and treatment have improved significantly. It is necessary to find novel biomarkers for reducing mortality rates 22. With the development of genomic analysis platform, more and more circRNA have been identified, several studies have proved that circRNA was closely associated with ccRCC 23, 24. In this study, we aimed to investigate the biological roles and mechanisms of circ-APBB1IP in ccRCC development.

A growing number of studies have indicated that circRNA has a vital regulatory function in ccRCC. For instance, Chen et al. reported that overexpression of hsa_circ_001895 promoted cell proliferation, invasion, migration, and inhibited cell apoptosis of ccRCC 25. Xue et al. indicated that circ-AKT3 inhibited migration and invasion of ccRCC via altering miR-296-3p/E-cadherin signals 26. Huang et al. revealed that circular RNA ABCB10 promoted cell proliferation and inhibits cell apoptosis of ccRCC 8. In the present study, we found that the expression of circ-APBB1IP was significantly elevated in ccRCC tissues and cells. Our results suggested that knockdown of circ-APBB1IP could suppress the proliferation, migration and invasion, whereas promoted the apoptosis in ccRCC cells. Deregulation of apoptosis was a hallmark of cancer and got related to tumor development and progression 27. Caspases are central to the mechanism of apoptosis; caspase-8 and caspase-9 are the initiators of apoptosis, while caspase-3 and caspase-7 act as executors 28. Irreparable genetic damage is one of the trigger of the initiation of the intrinsic mitochondrial pathway, which is strictly regulated by proteins belonging to the Bcl-2 family 29. In addition, the expression of cadherin and MMPs was closely associated with cell migration and invasive behavior 30. We have detected the expression of the above-related proteins. These results revealed that circ-APBB1IP might be an oncogenic factor in ccRCC cells.

At present, the mechanism of circRNA has been mainly reported as a molecular sponge for miRNA; however, the signaling pathway network was another essential mechanism of circRNA 31. For instance, circRNA has been identified to interact with Wnt/β-catenin pathway and AKT/mTOR signaling pathway in cancer by modulating key molecules of these pathway 32, 33. ERK1/2 is an important subfamily of MAPK that involve in a series of cellular activities and physiological processes 34. The activation of ERK1/2 regulates the activity of many transcription factors via phosphorylation, eventually influencing the specific biological effects of cells. In addition, the expression level of p-ERK1/2 is closely associated with progression of various cancers 35. In this study, we explored the critical protein of the ERK1/2 signaling pathway. The result showed that the expression of p-ERK1/2 was suppressed after the silencing of circ-APBB1IP. Hence, circ-APBB1IP may be as a regulator of ERK1/2 and induced ERK1/2 activation. Besides, the correlation of circRNA and ERK1/2 signaling pathway has been reported in the previous study 31. Therefore, there was reason to believe that circ-APBB1IP promoted cells growth, migration, invasion, and inhibited cells apoptosis via the ERK1/2 signaling pathway.

## Conclusion

In summary, this study was the first study to investigate the role and mechanism of circ-APBB1IP in ccRCC. Our results suggested that circ-APBB1IP was overexpressed in ccRCC tumor tissues and ccRCC cell lines, and circ-APBB1IP promoted ccRCC cells growth, migration, and invasion and suppressed apoptosis via ERK1/2 signaling pathway. These findings provided a potential target for molecular targeted therapy for ccRCC.

## References

[1] Du Y, Wang Q, Zhang X (2017). Lysophosphatidylcholine acyltransferase 1 upregulation and concomitant phospholipid alterations in clear cell renal cell carcinoma. J Exp Clin Cancer Res.

[2] Ljungberg B, Cowan NC, Hanbury DC (2010). EAU guidelines on renal cell carcinoma: the 2010 update. Eur Urol.

[3] Li JK, Chen C, Liu JY (2017). Long noncoding RNA MRCCAT1 promotes metastasis of clear cell renal cell carcinoma via inhibiting NPR3 and activating p38-MAPK signaling. Mol Cancer.

[4] Liang HF, Zhang XZ, Liu BG (2017). Circular RNA circ-ABCB10 promotes breast cancer proliferation and progression through sponging miR-1271. Am J Cancer Res.

[5] Hsiao KY, Sun HS, Tsai SJ (2017). Circular RNA - New member of noncoding RNA with novel functions. Exp Biol Med (Maywood).

[6] Jeck WR, Sharpless NE (2014). Detecting and characterizing circular RNAs. Nat Biotechnol.

[7] Wei Z, Chang K, Fan C (2019). Hsa_circ_0042666 inhibits proliferation and invasion via regulating miR-223/TGFBR3 axis in laryngeal squamous cell carcinoma. Biomed Pharmacother.

[8] Huang Y, Zhang Y, Jia L (2019). Circular RNA ABCB10 promotes tumor progression and correlates with pejorative prognosis in clear cell renal cell carcinoma. Int J Biol Markers.

[9] Yao J, Zhang C, Chen Y (2019). Downregulation of circular RNA circ-LDLRAD3 suppresses pancreatic cancer progression through miR-137-3p/PTN axis. Life Sci.

[10] Lu H, Yao B, Wen X (2019). FBXW7 circular RNA regulates proliferation, migration and invasion of colorectal carcinoma through NEK2, mTOR, and PTEN signaling pathways in vitro and in vivo. BMC Cancer.

[11] Wang S, Hu Y, Lv X (2019). Circ-0000284 arouses malignant phenotype of cholangiocarcinoma cells and regulates the biological functions of peripheral cells through cellular communication. Clin Sci (Lond).

[12] Mei M, Wang Y, Li Z (2019). Role of circular RNA in hematological malignancies. Oncol Lett.

[13] Li L, Zhang Z T (2020). Hsa_circ_0086414 Might Be a Diagnostic Biomarker of Oral Squamous Cell Carcinoma. Med Sci Monit.

[14] Livak K J, Schmittgen T D (2001). Analysis of relative gene expression data using real-time quantitative PCR and the 2(-Delta Delta C(T)) Method. Methods.

[15] Crowley LC, Waterhouse NJ (2016). Detecting Cleaved Caspase-3 in Apoptotic Cells by Flow Cytometry. Cold Spring Harb Protoc. 2016.

[16] Ola MS, Nawaz M, Ahsan H (2011). Role of Bcl-2 family proteins and caspases in the regulation of apoptosis. Mol Cell Biochem.

[17] Derycke LD, Bracke ME (2004). N-cadherin in the spotlight of cell-cell adhesion, differentiation, embryogenesis, invasion and signalling. Int J Dev Biol.

[18] Zhao Y, Tang H, Zeng X (2018). Resveratrol inhibits proliferation, migration and invasion via Akt and ERK1/2 signaling pathways in renal cell carcinoma cells. Biomed Pharmacother.

[19] Wang YN, Zhang LL, Fan XY (2018). Poly-L-Arginine Induces Apoptosis of NCI-H292 Cells via ERK1/2 Signaling Pathway. J Immunol Res.

[20] Gao Q, Gu Y, Jiang Y (2018). Long non-coding RNA Gm2199 rescues liver injury and promotes hepatocyte proliferation through the upregulation of ERK1/2. Cell Death Dis.

[21] Xu H, Zhao H, Yu J (2018). HOXB5 promotes retinoblastoma cell migration and invasion via ERK1/2 pathway-mediated MMPs production. Am J Transl Res.

[22] Wan B, Liu B, Yu G (2019). Differentially expressed autophagy-related genes are potential prognostic and diagnostic biomarkers in clear-cell renal cell carcinoma. Aging (Albany NY).

[23] Franz A, Ralla B, Weickmann S (2019). Circular RNAs in Clear Cell Renal Cell Carcinoma: Their Microarray-Based Identification, Analytical Validation, and Potential Use in a Clinico-Genomic Model to Improve Prognostic Accuracy. Cancers (Basel).

[24] Ma C, Qin J, Zhang J (2020). Construction and analysis of circular RNA molecular regulatory networks in clear cell renal cell carcinoma. Mol Med Rep.

[25] Chen Z, Xiao K, Chen S (2020). Circular RNA hsa_circ_001895 serves as a sponge of microRNA-296-5p to promote clear cell renal cell carcinoma progression by regulating SOX12. Cancer Sci.

[26] Xue D, Wang H, Chen Y (2019). Circ-AKT3 inhibits clear cell renal cell carcinoma metastasis via altering miR-296-3p/E-cadherin signals. Mol Cancer.

[27] Pistritto G, Trisciuoglio D, Ceci C (2016). Apoptosis as anticancer mechanism: function and dysfunction of its modulators and targeted therapeutic strategies. Aging (Albany NY).

[28] Thornberry N A, Lazebnik Y (1998). Caspases: enemies within. Science.

[29] Wong R S (2011). Apoptosis in cancer: from pathogenesis to treatment. J Exp Clin Cancer Res.

[30] Yeung K T, Yang J (2017). Epithelial-mesenchymal transition in tumor metastasis. Mol Oncol.

[31] Jiang Y, Zhang Y, Chu F (2020). Circ_0032821 acts as an oncogene in cell proliferation, metastasis and autophagy in human gastric cancer cells in vitro and in vivo through activating MEK1/ERK1/2 signaling pathway. Cancer Cell Int.

[32] Li Y F, Zhang J, Yu L (2019). Circular RNAs Regulate Cancer Onset and Progression via Wnt/beta-Catenin Signaling Pathway. Yonsei Med J.

[33] Xing L, Zhang L, Feng Y (2018). Downregulation of circular RNA hsa_circ_0001649 indicates poor prognosis for retinoblastoma and regulates cell proliferation and apoptosis via AKT/mTOR signaling pathway. Biomed Pharmacother.

[34] Lu Z, Xu S (2006). ERK1/2 MAP kinases in cell survival and apoptosis. IUBMB Life.

[35] Guo Y J, Pan W W, Liu S B (2020). ERK/MAPK signalling pathway and tumorigenesis. Exp Ther Med.

